# Supplementary material for: The Impact of Storage Temperature on the Development of Microbial Communities on the Surface of Blueberry Fruit
Source: Foods. 2023 Apr 11;12(8):1611. doi: 10.3390/foods12081611 (PMC10137657; doi:10.3390/foods12081611)
Supplement: Supplementary file 1 [file foods-12-01611-s001.zip › foods-2261235-supplementary.pdf]

Supplementary Materials

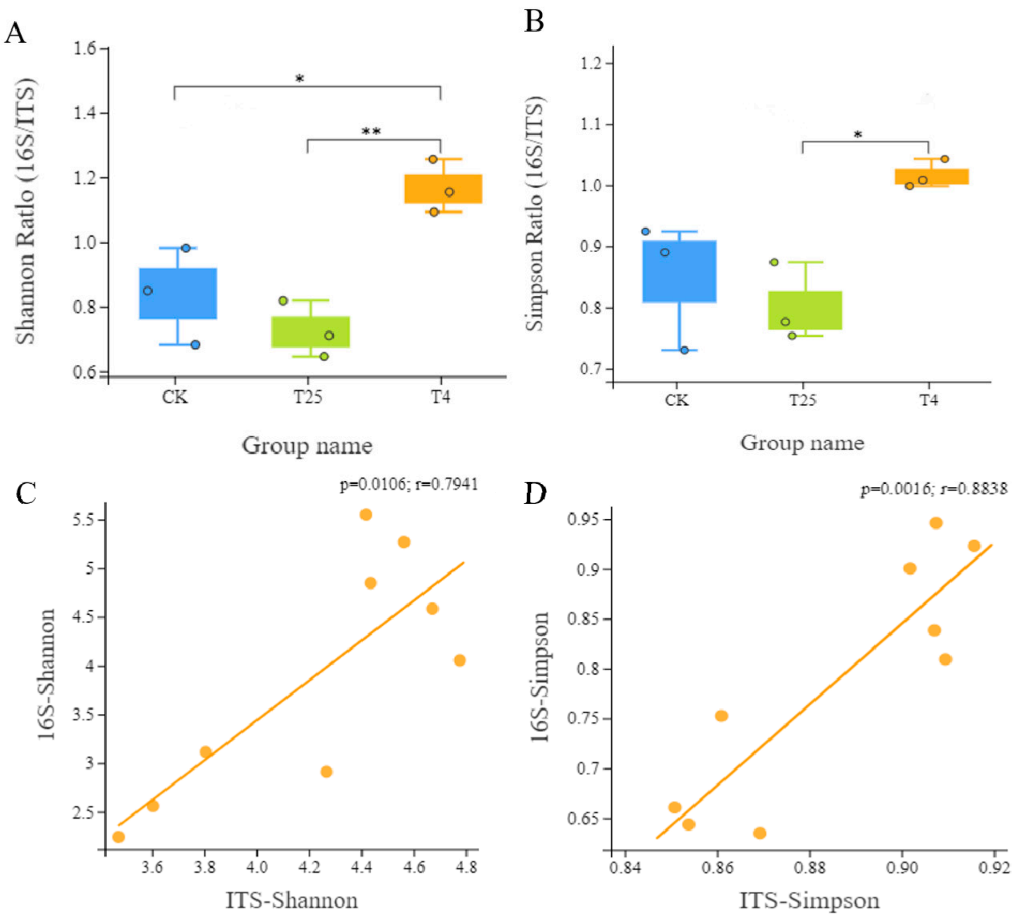

Figure S1. Scatter diagram of ratio difference between 16S and ITS Shannon and Simpson diversity indexes (*$p < 0.05$, **$p < 0.01$).

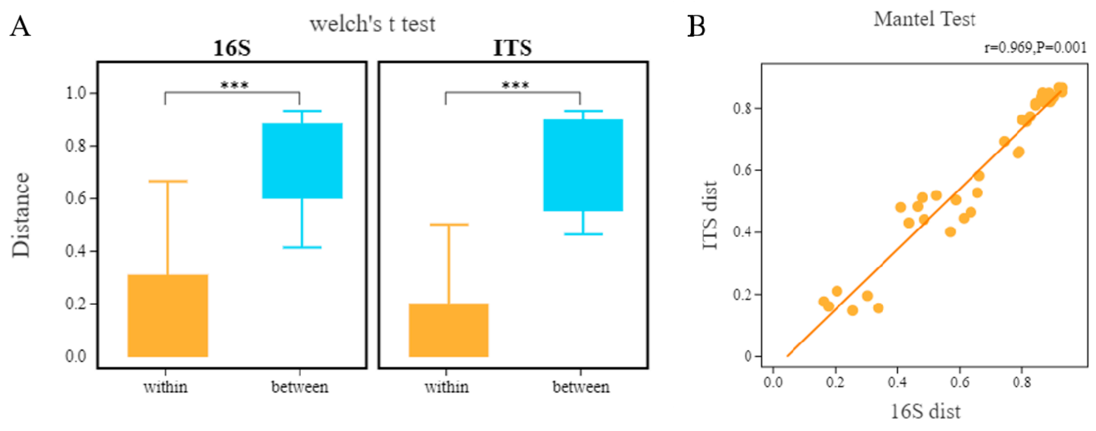

Figure S2. A and B for box plots of α-diversity index of bacterial and fungal microbial communities Shannon and Simpson index intra- and inter-group validation. C and D for scatter plot of distance between two pairs of ITS and 16S sequencing data (***$p < 0.001$).

Table S1. Statistical results of bacterial and fungal alpha diversity.

Items	No.	Name	CK_1	CK_2	CK_3	T_{25-1}	T_{25-2}	T_{25-3}	T_{4-1}	T_{4-2}	T_{4-3}
16S-Phylum	1	*Proteobacteria*	42.2976	39.5087	29.6582	87.1559	89.9268	92.7928	64.3013	59.3711	68.72
	2	*Cyanobacteria*	45.2418	43.8393	62.6897	4.3805	4.069	5.6399	10.8337	20.8681	18.4254
	3	*Actinobacteria*	3.7695	5.9862	2.2106	7.3402	5.329	0.9596	13.4127	10.895	5.9214
	4	*Bacteroidetes*	2.3239	3.1058	2.5415	0.231	0.1876	0.1418	4.6077	3.1042	1.6206
	5	*Firmicutes*	0.8725	4.7937	1.232	0.3854	0.0912	0.1008	2.4311	1.7806	0.8234
	6	*Planctomycetes*	0.0926	0.3142	0.0784	0.3664	0.1552	0.0171	1.9241	1.6588	0.8767
	7	*Acidobacteria*	0.3126	0.0992	0.1817	0.0466	0.0239	0.0186	1.0109	0.5959	0.7232
	8	*Armatimonadetes*	0.3548	0.1906	0.0033	0.0086	0.0068	0.0054	0.4556	0.2879	0.4433
	9	*Fusobacteria*	0.0099	0.0418	0.1117	0.0043	0.1347	0.145	0.0684	0.1258	1.1305
	10	*Patescibacteria*	0.0463	0.5144	0.1375	0.0138	0.0026	0.0046	0.2505	0.0393	0.0479
	11	*Deinococcus-Thermus*	0.1472	0.0009	0.0992	0.0129	0.0119	0.0496	0.0684	0.146	0.1035
	12	*Chlamydiae*	0	0.0017	0.0008	0.0103	0	0.0078	0.179	0.0604	0.0338
	13	*Verrucomicrobia*	0.0504	0.0009	0.0033	0.0034	0.0009	0	0.0201	0.1168	0.1078
	14	*Tenericutes*	0	0	0	0	0	0.0023	0.001	0	0.1851
	15	*Euryarchaeota*	0	0.1819	0	0	0	0	0	0	0.0152
	16	*Dadabacteria*	0	0.1062	0	0	0.0009	0.0008	0	0	0.0011
ITS-Phylum	1	*Ascomycota*	74.5048	70.5032	73.7686	85.1438	73.1386	89.3429	56.3058	60.8163	57.4251
	2	*Basidiomycota*	6.6453	2.6904	2.7312	7.0561	22.1275	6.2362	2.4357	3.6134	2.6295
	3	*Anthophyta*	5.7861	8.5481	9.7373	2.5767	0.7604	0.4166	2.8005	3.0943	4.0097

	4	*Chlorophyta*	0.7861	1.2581	0.7471	0.1476	0.0288	0.0593	2.5753	0.7655	0.8257
16S-Genus	1	*Gluconobacter*	0.7212	0.7765	0.5251	57.2579	9.8848	40.5434	2.0207	4.4842	3.1039
	2	*Methylobacterium*	8.3057	7.3319	7.8755	2.5993	2.8832	0.8953	19.5391	19.9853	23.5082
	3	*1174-901-12*	7.9451	6.5198	7.5963	2.0062	0.9395	0.6488	15.5812	13.6087	20.6482
	4	*Sphingomonas*	3.1336	2.473	1.117	0.3707	0.2617	0.2108	4.3894	3.9729	3.5276
	5	*Burkholderia-Caballeronia-Paraburkholderia*	4.1293	7.9996	2.9808	0.3173	0.2711	0.2325	0.8821	0.5415	0.9867
	6	*Hymenobacter*	0.9411	1.4519	0.1209	0.0655	0.0639	0.0279	2.4029	1.2028	0.428
	7	*Bdellovibrio*	0.5392	0.4178	0.0834	0.0681	0.1662	0.0248	1.5369	1.0398	1.0738
	8	*Tatumella*	0.0686	0.0287	0.02	0.0379	0.0477	0.0426	3.7095	0.462	0.1797
	9	*Massilia*	1.095	1.0332	0.2742	0.0578	0.0725	0.0659	3.1885	0.8173	0.7602
	10	*Methylocella*	0.1348	0.0357	0.1442	0.1638	0.0315	0.0357	1.6234	0.5365	1.4354
	11	*Acidiphilium*	1.0156	0.4422	0.8661	0.0974	0.1236	0.1124	1.0219	0.914	0.8419
	12	*Jatrophihabitans*	0.1232	0.1436	0.0125	0.0914	0.3708	0.0341	0.7343	0.7942	0.8582
	13	*Kineococcus*	0.1398	0.3395	0.135	0.0741	0.0631	0.0295	0.3209	0.2406	0.2647
	14	*Spirosoma*	0.8998	0.0157	0.0058	0.031	0.0435	0.0209	0.4094	0.459	0.3855
	15	*Geodermatophilus*	0.0157	0.0174	0.0117	0.0362	0.046	0.0163	0.2504	0.3291	0.1841
	16	*Vibrio*	0.1902	1.0411	0.005	0.0448	0.0094	0.7154	1.1869	0.0272	0.244
	17	*Staphylococcus*	0.2068	0.0122	0.2051	0.2845	0.0358	0.024	1.8598	0.8989	0.1688
	18	*Acinetobacter*	0.8568	1.747	0.1184	0.4156	0.0725	0.1318	0.1308	0.1832	0.2135
	19	*Singulisphaera*	0.0116	0.0044	0.0183	0.0897	0.0529	0.0085	0.5673	0.6442	0.4585
	20	*Novosphingobium*	0.0637	0.0165	0.02	0.025	0.0315	0.0163	0.1941	0.158	0.0893
	21	*Terriglobus*	0.0901	0.0514	0.1509	0.0362	0.0171	0.0109	0.7584	0.2204	0.5946
	22	*Cystobacter*	0.0157	0.0122	0.0092	0.0276	0.0153	0.0248	0.4124	0.9311	1.6304
	23	*Streptococcus*	0.3085	1.1116	0.496	0.0664	0.0247	0.0341	0.1237	0.3563	0.2505

24	*Mucilaginibacter*	0.0595	0.0061	0.0058	0.0854	0.0077	0.0062	1.1919	1.0629	0.1699
25	*Actinomycetospora*	0.0058	0.0113	0.005	0.1086	0.4186	0.014	0.4768	0.3312	0.085
26	*Quadrisphaera*	0.0504	0.497	0.0042	0.0276	0.0213	0.0085	0.1358	0.0564	0.0468
27	*Labrys*	0.0447	0.0052	0.015	0.031	0.0332	0.0209	0.0432	0.0584	0.025
28	*Bacillus*	0.1513	0.988	0.1734	0.0129	0.0068	0.0085	0.172	0.2949	0.1492
29	*Cetobacterium*	0.0091	0.0418	0.1117	0.0043	0.1347	0.145	0.0684	0.1117	1.1305
30	*Pantoea*	0.0835	0.007	0.1659	0.019	0.0102	0.0085	0.4627	0.0876	0.0229
31	*Dysgonomonas*	0.0074	0.0061	2.0539	0.0052	0.0017	0.0101	0.008	0.0141	0.0044
32	*Brachybacterium*	0.0579	0.0078	0.0058	0.0069	0.9224	0.0426	0.006	0.0262	0.1067
33	*Lapillicoccus*	0.0058	0.1541	0.0033	0.1828	0.1373	0.0093	0.0634	0.0262	0.0828
34	*Bryocella*	0.1447	0.0026	0	0.006	0.0043	0.0062	0.0825	0.156	0.0817
35	*Pseudomonas*	0.2861	0.4622	0.105	0.019	0.0196	0.0256	0.0956	0.1973	0.1939
36	*Enhydrobacter*	0.0033	0.8191	0.0625	0.119	0.0205	0.014	0.0573	0.0846	0.0958
37	*Kocuria*	0.0893	0.0061	0.1184	0.1121	0.0486	0.0202	0.0956	0.0936	0.1993
38	*Lactobacillus*	0.0637	0.3987	0.0308	0.0009	0.0077	0.0054	0.0372	0.0473	0.0577
39	*Deinococcus*	0.1472	0.0009	0.0992	0.0129	0.0119	0.0496	0.0684	0.1449	0.1035
40	*Nocardioides*	0.0728	0.2472	0.0392	0.0147	0.0136	0.0093	0.0563	0.0262	0.0163
41	*Exiguobacterium*	0.0033	0.726	0.005	0.0069	0.0026	0.0031	0.0694	0.0584	0.0316
42	*Corynebacterium_1*	0.005	0.3752	0.1642	0.0052	0.0077	0.0046	0.0553	0.0574	0.086
43	*Photobacterium*	0	0.7608	0	0.0052	0.0009	0.0031	0.0895	0	0.0098
44	*Caedibacter*	0.0017	0.0009	0.0375	0.0043	0.0009	0.0085	0.1116	0.1359	0.1405
45	*Cutibacterium*	0.0686	0.0157	0.1876	0.0147	0.006	0.0039	0.0272	0.0664	0.0272
46	*Marmoricola*	0.0033	0.0418	0	0.0957	0.0119	0.0031	0.2354	0.1047	0.0087
47	*Paracoccus*	0.1935	0.175	0.0358	0.0121	0.0102	0.0039	0.0191	0.0181	0.0773
48	*Amnibacterium*	0.0314	0.0409	0.0317	0.0466	0.0614	0.0225	0.0704	0.0533	0.0392
49	*Friedmanniella*	0.0695	0.0705	0.0025	0.0103	0.0196	0.0031	0.0785	0.159	0.0316
50	*Tanticharoenia*	0.0141	0.0157	0.0075	0.0293	0.0827	0	0.0352	0.0362	0.0207
51	*Aureimonas*	0.0017	0.0044	0.0017	0.1552	0.006	0.0062	0.008	0.1862	0.0653

52	*Psychroglaciecola*	0.0025	0.0026	0.0008	0.0112	0.0119	0.0078	0.0201	0.0805	0.0109
53	*Geobacter_sp_enrichment_culture_clone_VanCtr1*	0.0008	0	0	0.0017	0.0026	0.0047	0.2002	0.1087	0.0381
54	*Aeromonas*	0.0025	0.0427	0.0008	0.0034	0.0477	0.1403	0.1338	0.1067	0.086
55	*Larkinella*	0.0008	0	0.0008	0	0	0.0023	0.4637	0.0221	0.0022
56	*Granulicella*	0.0372	0	0	0.0026	0.0009	0.0008	0.1348	0.0292	0.0185
57	*Thermobrachium*	0.0521	0.3256	0.0383	0.0052	0.0009	0.0023	0.007	0.005	0.0033
58	*Conexibacter*	0.0008	0.0009	0.0008	0.0052	0.0017	0.0008	0.0191	0.0282	0.0566
59	*Tepidisphaera*	0	0.0026	0	0	0.0026	0	0.0131	0.1057	0.0142
60	*Chryseobacterium*	0.0984	0.0897	0	0.0078	0.006	0.0186	0.0091	0.0252	0.0392
61	*Kytococcus*	0.0008	0.2916	0	0.0009	0.0026	0.0093	0.005	0.002	0.0305
62	*Rothia*	0.0017	0.2873	0.0333	0	0.0017	0	0.0111	0.0493	0.0131
63	*Enterococcus*	0	0.1384	0.03	0.0009	0.0068	0.0008	0.005	0	0.0022
64	*Brevibacterium*	0	0.1219	0.0734	0.0009	0.0017	0.0333	0.0211	0.003	0.0087
65	*Rhodococcus*	0.1282	0.0017	0.0692	0.0043	0.0009	0.0046	0.008	0.0312	0.0065
66	*Chthoniobacter*	0	0.0009	0	0.0026	0.0009	0	0.0191	0.0695	0
67	*Mycobacterium*	0	0	0.0008	0.0086	0.0239	0	0.0211	0.1248	0
68	*Stenotrophomonas*	0.1381	0.0009	0.0017	0.025	0.0017	0.0202	0.003	0.0101	0.0011
69	*Cytophaga*	0	0.2124	0.0684	0	0.0034	0.0023	0.002	0.001	0.0011
70	*Belnapia*	0.0008	0.0566	0	0.0043	0.0026	0.0039	0.0101	0.0232	0.0044
71	*Candidatus_Paracaedibacter*	0	0	0	0.0009	0.0034	0	0.0111	0.0423	0
72	*Bradyrhizobium*	0.0017	0.1184	0.015	0.0052	0.0034	0.0039	0.007	0.0181	0.0087
73	*Siphonobacter*	0	0	0	0.0017	0	0	0.001	0.1419	0.0904
74	*Symplocastrum_CPER-KK1*	0.0008	0.2211	0.0017	0.0017	0	0	0.001	0.002	0.0011
75	*Mycoplasma*	0	0	0	0	0	0.0023	0.001	0	0.1851
76	*Bryobacter*	0	0	0	0	0	0.0008	0.004	0.1621	0.0283
77	*Leuconostoc*	0.0008	0	0.0767	0	0	0	0	0	0
78	*Salinicoccus*	0.0017	0.1811	0	0	0	0.0016	0	0	0.0022

	79	*Asinibacterium*	0	0.1819	0	0.0009	0.0017	0.0023	0	0	0.0011
	80	*Aerococcus*	0.0008	0.1314	0	0	0	0	0.008	0.002	0.0022
	81	*Salinicola*	0	0.1341	0.0358	0.0043	0	0.0023	0.001	0	0.0011
	82	*Prevotella_1*	0	0.0026	0	0	0	0	0.005	0	0.1666
	83	*Acidovorax*	0	0.1193	0.0008	0.0026	0.0017	0	0.0171	0.0151	0.012
	84	*Anaerococcus*	0	0.1219	0	0.0009	0	0.0008	0.001	0.0081	0.0272
	85	*Carnobacterium*	0	0.1436	0.0008	0	0	0.0008	0	0.001	0.0011
	86	*Jeotgalibaca*	0.0008	0.1462	0	0	0	0.0008	0	0	0
	87	*Sungkyunkwania*	0	0.1097	0	0.0009	0	0	0	0.002	0.0229
	88	*Candidatus_Alysiosphaera*	0.0017	0	0	0.0009	0.0017	0.0016	0.001	0.1158	0.0076
	89	*Actinomyces*	0	0.1088	0	0.0034	0	0	0.004	0.0121	0.0011
	90	*Dietzia*	0.1174	0	0	0.0009	0	0	0	0	0
	91	*Catalinimonas*	0	0.1149	0	0.0017	0	0	0	0	0
	92	*Hanstruepera*	0.0008	0.1027	0	0	0	0	0	0	0
	1	*Zasmidium*	4.318	3.6118	3.6054	20.734	10.0557	5.4232	17.0975	12.7426	15.4657
	2	*Talaromyces*	0.3911	0.7418	0.3577	32.6347	31.0375	23.4981	1.1929	1.2013	1.0631
	3	*Aspergillus*	1.5129	1.8517	5.356	9.158	20.873	47.4784	1.2741	1.5311	3.3888
ITS-Genus	4	*Uwebraunia*	25.1654	24.7643	31.8735	0.5276	0.4653	0.4052	7.3601	6.741	5.4366
	5	*Vaccinium*	5.6723	8.1027	9.5227	2.5558	0.7454	0.2424	2.755	3.0609	3.9715
	6	*Alternaria*	3.1543	4.1218	4.0177	0.8339	0.6516	0.4128	4.4211	7.7851	4.0805
	7	*Strelitziana*	3.1154	4.4577	3.666	0.6838	0.5728	0.4923	5.0197	4.0372	5.9159
	8	*Colletotrichum*	0.359	0.0503	0.6021	1.5755	2.6928	6.4685	0.0844	0.1452	0.2691
	9	*Coniophora*	0.0117	0.0763	0.0139	0.2386	16.8157	0.4279	0.0043	0.4441	0.0098

10	*Aureobasidium*	7.2028	4.5978	4.9755	0.048	0.0513	0.0896	0.7285	1.0941	0.5436
11	*Phaeophleospora*	0.4583	0.6537	0.5981	0.4797	0.5891	0.4393	1.9475	1.7466	1.9705
12	*Irpex*	0.2413	0.0314	0.0119	0.1033	0.1088	5.1531	0.0108	0.006	0.1111
13	*Neofusicoccum*	0.0234	0.0224	0.3746	0.0074	0.0063	0.0063	0.0065	0.0131	0.0033
14	*Botryosphaeria*	0.2355	0.3763	0.0268	0.0283	0.0263	0.0063	0.0119	0.0155	0.0109
15	*Schizophyllum*	0.108	0.0054	0.0129	5.5592	0.5803	0.2133	0.1602	0.0179	0.0207
16	*Passalora*	1.2668	4.4424	2.0347	0.0738	0.0513	0.053	0.6582	0.9286	0.7189
17	*Candida*	0.0778	0.0045	0.0666	0.0713	0.0088	0.125	0.2587	0.2548	0.085
18	*Erythrobasidium*	1.7007	0.9321	0.8743	0.0357	0.0575	0.0467	0.4774	1.0298	0.7244
19	*Neocladophialophora*	0.1265	0.123	0.0387	0.0787	0.07	0.0745	0.6441	0.2191	0.3072
20	*Starmerella*	0	0.0018	0.004	5.7277	0.0163	0.0227	0.0779	0.0036	0
21	*Ascochyta*	0.4544	0.4894	0.4282	0.043	0.0425	0.0505	0.6636	0.5893	0.4989
22	*Sphaerulina*	1.5509	1.3335	0.91	0.0037	0.0088	0.0126	0.1212	0.1833	0.1492
23	*Papiliotrema*	0.2608	0.2416	0.1778	0.0726	0.05	0.0644	0.5759	0.4977	0.4858
24	*Zygoascus*	0	0.0009	0	4.0612	0.1026	0.0619	0.0639	0.0048	0.0044
25	*Pestalotiopsis*	0.1012	0.0153	0.0358	0.1181	0.1351	0.6325	0.053	0.181	0.1089
26	*Camptophora*	0.4845	0.5011	0.2623	0.032	0.0263	0.029	0.5304	0.0583	0.0839
27	*Dioszegia*	0.1703	0.0673	0.1262	0.0307	0.0188	0.0379	0.367	0.4084	0.5305
28	*Paraconiothyrium*	0.7696	0.3951	0.308	0.0332	0.0075	0.0101	0.144	0.1191	0.1133
29	*Neopestalotiopsis*	0.2209	0.0907	0.0179	0.0197	0.0113	0.0114	0.0292	0.0845	0.0534
30	*Phlebiopsis*	0	0.0467	0	0	0.0013	0	0.0011	0	0
31	*Cryptococcus*	1.2434	0.2694	0.1272	0.0049	0.005	0.005	0.1396	0.1107	0.0708
32	*Pichia*	0.0068	0.0009	0	0.6027	1.2845	0.0518	0.0097	0.0238	0.0185
33	*Golubevia*	0.1946	0.0548	0.1073	0.032	0.0375	0.0164	0.131	0.3298	0.1307
34	*Peniophora*	0.036	0	0.006	0.0271	0.0075	0.0076	0.0097	0	0.0109
35	*Pseudocercospora*	0.2452	0.1293	0.1917	0.0234	0.0213	0.0076	0.0498	0.1	0.0664
36	*Rhodosporidiobolus*	1.1627	0.1275	0.0477	0	0	0	0.0141	0.025	0.0294
37	*Trebouxia*	0.0681	0.5855	0.155	0.0123	0.0025	0.0063	0.2317	0.0512	0.0545

38	*Zygosaccharomyces*	0	0.0009	0.0179	0.0025	0.0063	0.0694	0.0011	0.0012	0.0044
39	*Hanseniaspora*	0.0146	0.0135	0.004	0.0086	0.454	0.0076	0.0314	0.1119	0.0675
40	*Ramichloridium*	0.0302	0.0314	0.0199	0.0148	0.0213	0.0177	0.0617	0.0167	0.0142
41	*Ramularia*	0.288	0.3538	0.1133	0.0037	0	0	0.0271	0.0167	0.0305
42	*Sporobolomyces*	0.2919	0.1392	0.304	0.0025	0.0013	0	0.0379	0.0441	0.0359
43	*Penicillium*	0.001	0	0.0159	0.3837	0.0113	0.0114	0.0119	0.0024	0
44	*Periconia*	0.0983	0.0952	0.0974	0.0111	0.0113	0.005	0.0963	0.1	0.0861
45	*Keissleriella*	0.0097	0.0153	0.0139	0.0062	0.0175	0.0114	0.0574	0.1691	0.1623
46	*Phyllozyma*	0.144	0.0323	0.4073	0.0037	0.0025	0	0.0292	0.0488	0.0229
47	*Leptospora*	0.0311	0.0314	0.1023	0.0111	0.0138	0.0088	0.0714	0.0667	0.0741
48	*Taphrina*	0.1099	0.0925	0.0318	0.0062	0.005	0.0076	0.0444	0.075	0.085
49	*Stagonospora*	0.0107	0.0144	0.0238	0.0086	0.0075	0.0063	0.0704	0.0786	0.1166
50	*Agriophyllum*	0.0107	0.1814	0.0378	0.0037	0.0063	0.1692	0.0022	0.0012	0.0163
51	*Dissoconium*	0.2179	0.1625	0.0318	0.0012	0	0.0013	0.0227	0.0226	0.0218
52	*Xenoacremonium*	0.0019	0	0.0238	0	0.0013	0.0025	0	0	0
53	*Diaporthe*	0.2092	0.0638	0.0278	0.0049	0.0125	0.0025	0.0162	0.0131	0.0087
54	*Sphaceloma*	0.0156	0.0539	0.153	0.0062	0.0013	0.0025	0.0227	0.0631	0.0185
55	*Filobasidium*	0.1197	0.0898	0.0646	0.0012	0.0013	0.0013	0.0097	0.0286	0.0076
56	*Pseudoveronaea*	0.0749	0.1338	0.0189	0	0	0	0.0087	0.0024	0.0109
57	*Anthracocystis*	0	0	0	0	0	0	0.0032	0	0.0044
58	*Meira*	0.1411	0.0099	0.0129	0	0	0	0.0011	0	0.0033
59	*Microstroma*	0.0068	0.1203	0.004	0	0	0	0	0.0095	0.0011

Table S2. Top 10 bacterial genera with the highest relative abundance in each group. Means with standard deviation are shown. Different lowercase letters (a, b, c) in the same line indicate significant difference ($p < 0.05$) of means between groups.

Group		CK (%)	T25 (%)	T4 (%)
Phylum	Proteobacteria	37.15 ± 3.83^{c}	89.96 ± 1.63^{a}	64.13 ± 2.70^{b}
	Cyanobacteria	50.59 ± 6.06^{a}	4.7 ± 0.48^{b}	16.71 ± 3.02^{b}
	Actinobacteria	3.99 ± 1.09^{b}	4.54 ± 1.88^{b}	10.08 ± 2.20^{a}
	Bacteroidetes	2.66 ± 0.23^{a}	0.19 ± 0.03^{b}	3.11 ± 0.86^{a}
	Firmicutes	2.30 ± 1.25^{a}	0.19 ± 0.096^{a}	1.68 ± 0.47^{a}
	Planctomycetes	$0.16 \pm 0.0.08^{b}$	$0.18 \pm 0.0.10^{b}$	$0.54 \pm 0.0.31^{a}$
	Acidobacteria	0.2 ± 0.06^{b}	0.03 ± 0.009^{b}	0.78 ± 0.12^{a}
	Armatimonadetes	0.18 ± 0.10^{ab}	0.0069 ± 0.00^{b}	0.40 ± 0.05^{a}
	Fusobacteria	0.055 ± 0.03^{a}	0.095 ± 0.045^{a}	0.445 ± 0.34^{a}
	Patescibacteria	0.23 ± 0.14^{a}	0.007 ± 0.00^{a}	0.11 ± 0.07^{a}
Genus	Gluconobacter	0.67 ± 0.08^{b}	35.9 ± 13.87^{a}	3.2 ± 0.71^{b}
	Methylobacterium	7.84 ± 0.28^{b}	2.13 ± 0.62^{c}	21.01 ± 1.26^{a}
	1174-901-12	7.35 ± 0.43^{b}	1.2 ± 0.41^{c}	16.61 ± 2.10^{a}
	Sphingomonas	2.24 ± 0.59^{b}	0.28 ± 0.05^{c}	3.96 ± 0.25^{a}
	Burkholderia-Caballeronia-Paraburkholderia	5.04 ± 1.52^{a}	0.27 ± 0.02^{b}	0.8 ± 0.13^{b}
	Hymenobacter	0.84 ± 0.39^{a}	0.05 ± 0.012^{a}	1.34 ± 0.57^{a}
	Bdellovibrio	0.35 ± 0.14^{b}	0.086 ± 0.04^{b}	1.22 ± 0.16^{a}
	Tatumella	0.039 ± 0.15^{a}	0.043 ± 0.00^{a}	1.45 ± 1.13^{a}
	Massilia	0.8008 ± 0.26^{a}	0.0654 ± 0.00^{a}	1.5887 ± 0.8^{a}
	Methylocella	0.1 ± 0.03^{b}	0.08 ± 0.04^{b}	1.2 ± 0.34^{a}

Table S3. Top 10 fungal genera with the highest relative abundance in each group. Means with standard deviation are shown. Different lowercase letters (a, b, c) in the same line indicate significant difference (p < 0.05) of means between groups.

Group		CK	T25	T4
Phylum	Ascomycota	72.93 ± 1.23^{a}	82.54 ± 4.86^{a}	58.18 ± 1.36^{b}
	Basidiomycota	4.02 ± 1.31^{b}	11.81 ± 5.17^{a}	2.89 ± 0.36^{b}
	Anthophyta	8.02 ± 1.17^{a}	1.25 ± 0.67^{b}	3.3 ± 0.36^{b}
	Chlorophyta	0.93 ± 0.16^{ab}	0.08 ± 0.04^{b}	1.39 ± 0.59^{a}
Genus	Zasmidium	3.85 ± 0.24^{b}	12.07 ± 4.53^{ab}	15.1 ± 1.27^{a}
	Talaromyces	0.5 ± 0.12^{b}	29.06 ± 2.82^{a}	1.15 ± 0.05^{b}
	Aspergillus	2.91 ± 1.23^{b}	25.84 ± 11.34^{a}	2.06 ± 0.67^{b}
	Uwebraunia	27.27 ± 2.31^{a}	0.47 ± 0.04^{c}	6.51 ± 0.57^{b}
	Vaccinium	7.77 ± 1.12^{a}	1.18 ± 0.70^{b}	3.26 ± 0.37^{b}
	Alternaria	3.76 ± 0.31^{a}	0.63 ± 0.12^{b}	5.43 ± 1.18^{a}
	Strelitziana	3.75 ± 0.39^{a}	0.58 ± 0.06^{b}	5.00 ± 0.54^{a}
	Colletotrichum	0.34 ± 0.16^{b}	3.58 ± 1.48^{a}	0.17 ± 0.05^{b}
	Coniophora	0.03 ± 0.021^{a}	5.83 ± 0.021^{a}	0.15 ± 0.15^{a}
	Aureobasidium	5.6 ± 0.81^{a}	0.06 ± 0.01^{b}	0.79 ± 0.16^{b}
